# Interplay of transport vesicles during plant-fungal pathogen interaction

**DOI:** 10.1007/s44154-023-00114-0

**Published:** 2023-08-22

**Authors:** Yakubu Saddeeq Abubakar, Idris Zubair Sadiq, Aarti Aarti, Zonghua Wang, Wenhui Zheng

**Affiliations:** 1 State Key Laboratory of Ecological Pest Control for Fujian and Taiwan Crops, College of Life Sciences, Fujian Agriculture and Forestry University, Fuzhou, China; 2 Key Laboratory of Integrated Pest Management for Fujian-Taiwan Crops, Ministry of Agriculture, College of Plant Protection, Fujian Agriculture and Forestry University, Fuzhou, China; 3 Department of Biochemistry, Faculty of Life Sciences, Ahmadu Bello University, Zaria, Nigeria; 4 Fuzhou Institute of Oceanography, Minjiang University, Fuzhou, China

**Keywords:** Endosomes, Extracellular vesicles, Plant-pathogen interaction, Phytopathogens, Vesicles trafficking

## Abstract

Vesicle trafficking is an essential cellular process upon which many physiological processes of eukaryotic cells rely. It is usually the ‘language’ of communication among the components of the endomembrane system within a cell, between cells and between a cell and its external environment. Generally, cells have the potential to internalize membrane-bound vesicles from external sources by endocytosis. Plants constantly interact with both mutualistic and pathogenic microbes. A large part of this interaction involves the exchange of transport vesicles between the plant cells and the microbes. Usually, in a pathogenic interaction, the pathogen releases vesicles containing bioactive molecules that can modulate the host immunity when absorbed by the host cells. In response to this attack, the host cells similarly mobilize some vesicles containing pathogenesis-related compounds to the pathogen infection site to destroy the pathogen, prevent it from penetrating the host cell or annul its influence. In fact, vesicle trafficking is involved in nearly all the strategies of phytopathogen attack subsequent plant immune responses. However, this field of plant-pathogen interaction is still at its infancy when narrowed down to plant-fungal pathogen interaction in relation to exchange of transport vesicles. Herein, we summarized some recent and novel findings unveiling the involvement of transport vesicles as a crosstalk in plant-fungal phytopathogen interaction, discussed their significance and identified some knowledge gaps to direct future research in the field. The roles of vesicles trafficking in the development of both organisms are also established.

## Introduction

**Fig. 1:**
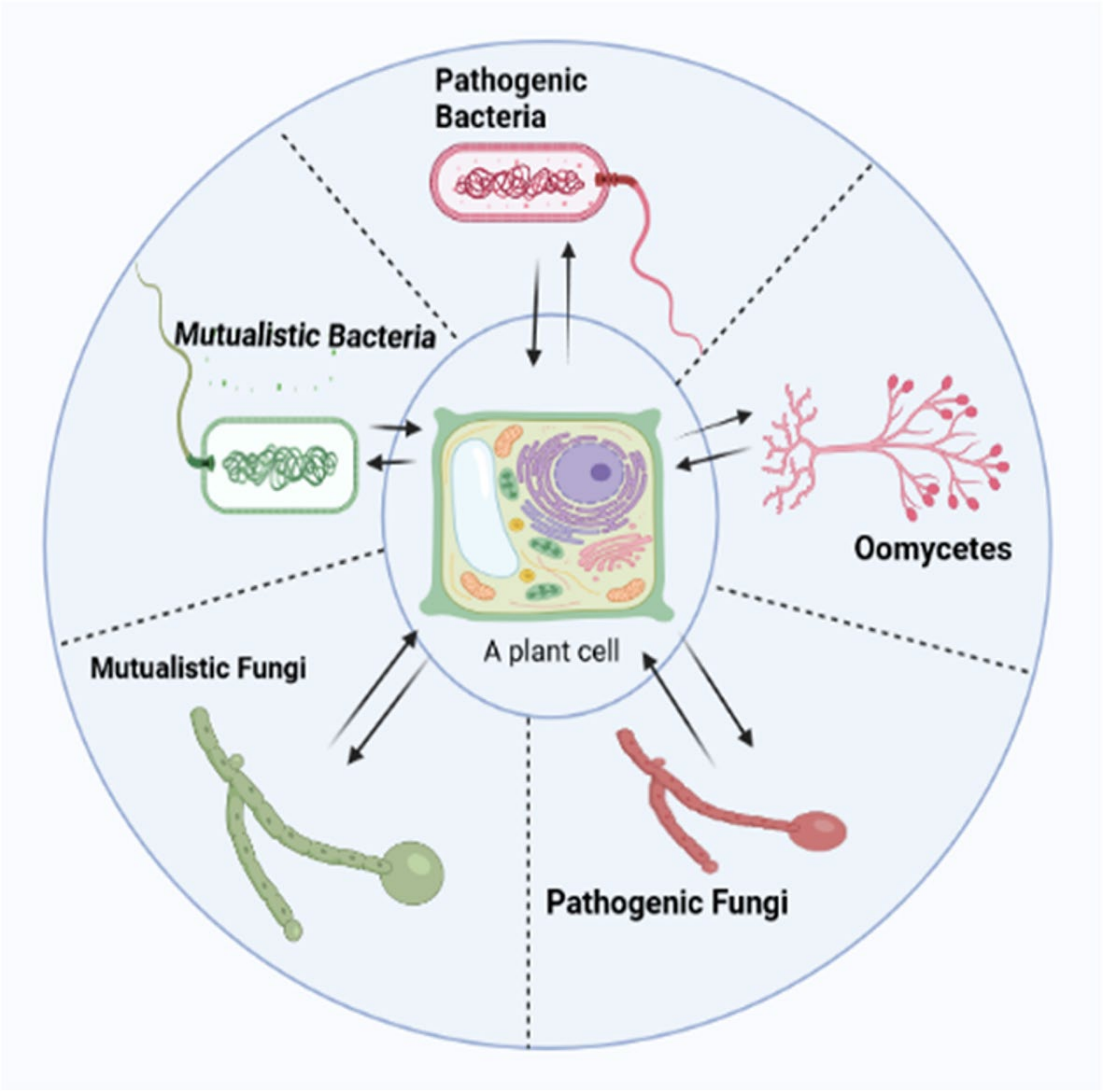
The various microorganisms that interact with plant cells. The interaction can be mutualistic or pathogenic. Pathogenic organisms are shown in red while mutualistic ones are shown in green

Being sessile, plants come in contact with many different microorganisms most of which establish mutualistic relationship with them while a few are pathogenic. Generally, plants lack specific immune cells; upon encounter with a microbe, a plant cell reprograms its physiologic processes to either defend itself or prepare for the establishment of a mutualistic relationship as the case may be (Ruano and Scheuring 2020). Although, it is not yet clearly understood how plants distinguish mutualistic from pathogenic microbes, it is known that pathogenic fungi have ergosterols on their cell surfaces, while mutualistic fungi have sterols instead (Jordá and Puig 2020). Plants associate with mutualistic bacteria or fungi. Association with mutualistic bacteria such as *Rhizobia* is achieved through symbiosome as an interfacial surface, surrounded by peribacteriod membrane (PBM). Periarbuscular membrane (PAM) however forms the interfacial surface for interaction with mutualistic fungi (mycorrhizal hypha) (Kanazawa and Ueda 2017). On the other hand, pathogenic microbes (fungi and most oomycetes) usually form haustoria which are surrounded by host-generated membrane called extrahaustorial membrane (EHM) that limits pathogen proliferation within the host cell (Berkey et al. 2017). Pathogenic bacteria (and some oomycetes) often use their secretion systems to ‘inject’ effector substances into the host cells. Figure 1 summarizes the various microorganisms that interact with plants.

Plants generally possess two levels of basal immunity: pattern-triggered immunity (PTI) and effector-triggered immunity (ETI). In PTI, when a pathogen comes in contact with the host plant, a given molecular signature on the pathogen known as pathogen associated molecular pattern (PAMP) is identified by the host plasma membrane-localized receptor proteins called pattern recognition receptors (PRRs) via interaction between the two (Chang et al. 2022). The interaction of a PAMP with a PRR causes phosphorylation (activation) of the latter and it consequently gets internalized for downstream signaling and subsequent degradation in the vacuole to avoid over-activation of the immune response (Couto and Zipfel 2016). PTI is a very effective defense line against many phytopathogens. However, there are some adapted pathogens that release certain effector proteins that block the operation of the PTI, rendering the plant susceptible to the pathogens as the effectors hijack the host cell functions to favor the pathogens’ colonization, and at the same time maintain the viability of the host cells (Toruño et al. 2016). To take care of these effectors, plants evolved the ETI line of innate immunity which involves the recognition and handling of pathogen effectors using intracellular family of protein receptors called nucleotide-binding leucine-rich repeat (NB-LRR) receptors (Gu et al. 2017; Park et al. 2018). This is a very strong response that can trigger programed cell death (PCD) of the infected cells. Plant innate immunity, whether PTI or ETI, generally depends on vesicle/membrane trafficking.

In plants, endomembrane system principally consists of the endoplasmic reticulum (ER), Golgi complex, trans-Golgi network (TGN), endosomes and vacuoles. Effective communication between these organelles and with the plasma membrane (PM) is very necessary for the continuity of cellular activities in both healthy and diseased conditions, and this is achieved through vesicle trafficking. This trafficking occurs for biosynthetic, recycling, degradative and/or defense purposes. It is central in maintaining physiologic homeostasis, signaling, growth and development of eukaryotic cells. It is also very important for the continuity of secretory and endocytic pathways. In plant cells, there are different vesicles trafficking routes including ER-TGN, ER-vacuole, TGN-endosomes, endosomes-vacuoles, TGN-PM, and many more. When a plant cell recognizes the presence of a microbial species (pathogenic or beneficial) around it, it reprograms its molecule homeostasis and endomembrane system in preparation for that encounter. This brings about changes in molecular architecture of the cell, including changes in lipid composition and the symmetry of the plasma membrane. Intracellular vesicle trafficking is also reshaped for appropriate responses. In fact, the entire process of host cell reprogramming heavily depends on vesicle trafficking. A number of previous studies demonstrated the significance of vesicle trafficking in plants during plant–microbe interaction. However, the link between vesicle trafficking in plants and that in phytopathogens during plant-pathogen interaction is not clearly documented. In the present review, we bring together some important findings to demonstrate how phytopathogens make use of their endogenous transport vesicles to promote virulence and manipulate host immunity and how the host plants use same to counteract, disarm and incapacitate the invading pathogens.

## Diversity of transport vesicles

**Fig. 2:**
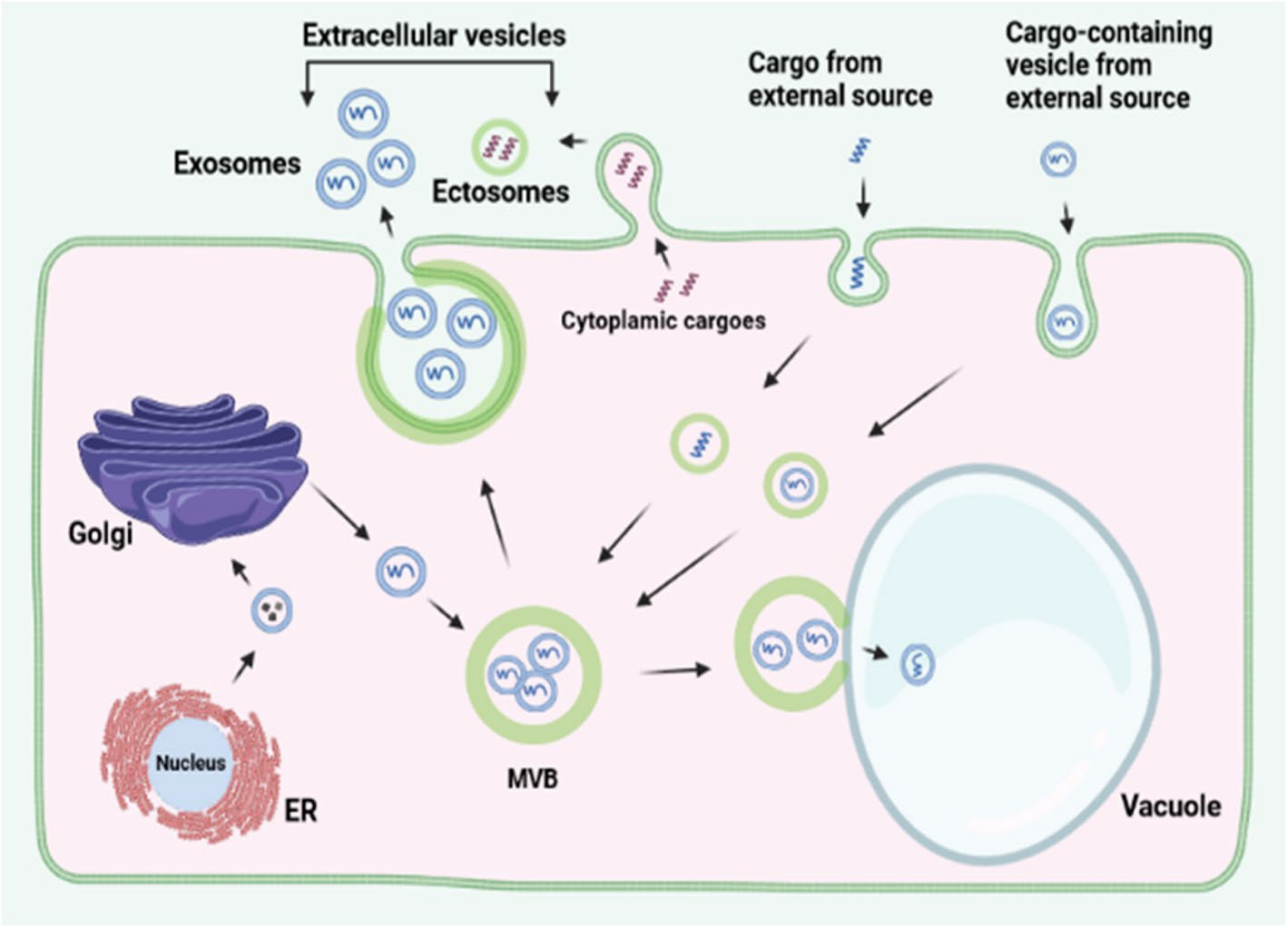
Some notable vesicle trafficking pathways in plant cells. Cargos from external sources are internalized in vesicles and integrated in a multivesicular body (MVB) for sorting and subsequent channeling to their appropriate destinations. After their synthesis at the endoplasmic reticulum (ER), proteins are packaged in transport vesicles and delivered to the Golgi complex. Cargos in the Golgi can be delivered to the MVB for further transport processes. Cytoplasmic cargos can be packaged in plasma membrane-derived vesicles and released to the extracellular space as ectosomes

Transport vesicles are membrane-bound, small sphere-like intracellular structures containing some selected cargo molecules to be delivered to a particular destination within or outside a eukaryotic cell for a given purpose. Such vesicles could be as small as 40 nm and as large as 10 µm in diameter (Vagner et al. 2019). Transport vesicles can be divided into intracellular and extracellular vesicles (EVs). Usually, intracellular vesicles are generated by endocytosis (e.g. endosomes) and the target destination is within the cell. However, internally generated vesicles that are delivered within the cell (such as ER-Golgi vesicles) are also considered as intracellular vesicles. Extracellular vesicles are generated from within the cell and delivered outside the cell. They are further divided into two based on their sites of biogenesis: exosomes and ectosomes (or microvesicles). The exosomes are smaller with a maximum diameter of 100 nm and are generated from the endosomal system and internalized into the lumen of late endosomes as intraluminal vesicles (ILVs), hence the late endosomes harboring the ILVs are called multivesicular bodies (MVBs). The MVBs are transported to the PM for subsequent release of the ILVs to the extracellular space by exocytosis (Abels and Breakefield 2016; U Stotz et al. 2022). However, MVBs may also deliver their contents to the vacuoles for degradation. What determine the specific pathway for MVB transportation still remain unclear. Ectosomes are generated at the PM following outward folding of the PM, enclosing the cargos, and subsequent budding off of the vesicles to the extracellular environment. They are more than 100 nm in diameter and emerge mainly from the PM for delivery to the extracellular environment (Fig. 2). The ER serves as the major source of vesicles for trafficking purposes. ER-Golgi vesicles are often coated with coatamer protein II (COPII) whereas the reverse transport (Golgi-ER) involves COPI-coated vesicles (Béthune and Wieland 2018; Robinson 2020). An important feature of COPII coated vesicles is that they are rich in lysophospholipids (Melero et al. 2018). Although the factors that determine cargo selectivity and correct targeting of vesicles still remain in the dark, TGN vesicles containing cargos that are destined for vacuolar degradation are clathrin-coated (Dahhan et al. 2022). Secreted proteins from the ER are packaged in certain vesicles at the TGN for subsequent shuttling to the PM. However, there are still some knowledge gaps on the TGN-PM trafficking route. Usually, there is balance between inward and outward vesicle secretion; in plants, however, outward secretion is most often associated with countering pathogen attack. It remains unproven how large extracellular vesicles traverse through the tight and rigid pores of fungal cell walls, though it was hypothesized that the lipid bilayers, being compressible, squeeze themselves through the pores (Casadevall et al. 2009).

## Vesicles biogenesis and trafficking events

The biogenesis of transport vesicles begins with donor membrane curvature, usually induced by BAR domain-containing proteins (Abubakar et al. 2017). Cargo recruitment and orchestration then begin in the curved membrane, and these are regulated by a number of proteins/protein complexes including phox homology domain containing proteins, SNX4/41/42, clathrin, retromer complex, and some SNARE proteins (Abubakar et al. 2017, 2021). However, the factors that determine cargo selection are still under investigation, although it is known that prior to ILV formation, the endosomal membrane becomes enriched with tetraspanins, especially CD9 and CD63 (U Stotz et al. 2022). This is accompanied by recruitment of endosomal sorting complexes required for transport (ESCRT) to the endosomal membrane for ILVs formation (Abels and Breakefield 2016). Generally, ESCRT has four different subcomplexes. ESCRT-0 is involved in cargo clustering, ESCRT-I and ESCRT-II function in bud formation while ESCRT-III is for vesicle formation/scission (Hanson et al. 2009; Oliveira et al. 2013). After exerting their functions, the various components of the ESCRT complexes dissociate and are recycled by some accessory proteins (Hanson et al. 2009). Following ILVs formation, MVBs are subsequently transported to the PM for fusion and release of the ILVs. There are many subcellular factors that mediate these processes at different levels. For instance, Rab-GTPases mediate intracellular vesicle trafficking at different stages, including vesicle budding, mobility on cytoskeleton track as well as fusion with the PM (Spang 2004). SNARE proteins are also indispensable for the fusion of vesicles with the PM.

AP2 complex is important for generation of vesicles and cargo selection (Xu et al. 2019). The additional role of this protein complex in the pathogenicity and polarized growth of *Fusarium graminearum*, a biotrophic phytopathogenic fungus that causes Fusarium head blight (FBH) of wheat and some cereals, further suggests a link between vesicle trafficking and virulence/development of phytopathogens (Zhang et al. 2019). Vesicle budding is mediated by ARF/AR1 (Wang et al. 2016). During plant-pathogen interaction, almost all vesicle transports involve RAB GTPases, including transport to infection sites and in effector binding (Spang 2004; Tripathy et al. 2021). The small GTPase Arf6 is a well-known regulator of vesicle trafficking during endocytosis (Zhu et al. 2016). BIGs serve as guanine-nucleotide exchange factors for ARF proteins, and vesicle trafficking requires the functions of *BIG3* and *BIG5* for the physiologic functions and development of Arabidopsis (Suo et al. 2021; Xue et al. 2019).

Nearly all intracellular vesicle transports involve motor proteins along the cytoskeletal tracks. There are many transport machineries that are involved in vesicle transport to different destinations but the factors that determine the choice of these machineries for a given vesicle are not very clear. Docking and tethering at the target membrane are regulated by RAB GTPases and tethering factors while SNARE proteins ensure the fusion of the vesicles with the target membrane (Wang et al. 2016;). In *Verticillium dahliae* (a vascular wilt pathogen), the SNARE proteins VdSso1 and VdSec22 regulate vesicle-mediated cargo delivery at the target membrane (J., Wang et al. 2018a, b). In *F. graminearum*, SNARE proteins regulate not only vesicle fusion but also the fungal developmental/virulence processes like deoxynivalenol (DON, a mycotoxin) production (Li et al. 2019). In addition to SNARE proteins, RAB GTPases were also shown to be involved in vesicle fusion. MoRab5A and MoRab5B regulate general endocytosis and the fusion of vesicles with the vacuole in *Magnaporthe oryzae* (Yang et al. 2017). FgSnc1-mediated vesicle fusion with the PM is regulated by the Rab8 GTPase activating protein FgMsb3 (Zheng et al. 2021a, b). The recycling of FgSnc1 for effective vesicle fusion is ensured by Snx41-Snx4 dimer in *F. graminearum* (Zheng et al. 2018a, b). Due to their intimate association with membranes, flippases also serve as key regulators of vesicle fusion, and blockage of membrane trafficking due to deletion of these enzymes also results in attenuated DON biosynthesis and consequently loss of virulence in *F. graminearum* (Oliveira et al. 2013; Yun et al. 2020). MoVps17, a subunit of the retromer complex, has also been shown to be indispensable for vesicle budding, fusion and endocytosis in *M. oryzae* (Wu et al. 2021; Zheng et al. 2017).

## Vesicles trafficking and development/virulence of phytopathogenic fungi

Vesicles trafficking is critically important for the growth, development as well as virulence of phytopathogenic fungi (Shoji et al. 2014). Some phytopathogic fungi have been shown to use transport vesicles to deliver growth factors to the fungal apex, necessary for their polarized growths. For example, the polarized growth and virulence of *F. graminearum* were observed to be impaired after blocking vesicle secretion to the PM by deletion of FgSnc1, a t-SNARE protein that coordinates the fusion of secreted vesicles with the PM (Qiu et al. 2019; Zheng et al. 2018a, b). Interfering with intracellular vesicle transport also negatively affects conidiation, conidial morphology, asexual reproduction and production of DON mycotoxin in *F. graminearum* (Adnan et al. 2020; Yang et al. 2020; Zheng et al. 2021a, b). Autophagy is an important physiologic process that regulates the intracellular homeostasis of many cytoplasmic components. Operation of autophagy requires the integrated functions of autophagic genes like *ATG9*. The general autophagy and pathogenicity of *F. graminearum* is largely dependent on effective vesicle-mediated trafficking of FgAtg9 to phagophore assembly site (Zheng et al. 2018a, b). Ede1p induce site initiation for endocytosis of vesicles in yeast, and this protein is important not only for autophagy but also virulence of *F. graminearum* (Han et al. 2020). The sorting and transport of cargo molecules depends largely on phox homology (PX) domain-containing proteins. Through their vesicles trafficking functions, these proteins control the growth, reactive oxygen species (ROS) production and virulence of *F. graminearum* (Lou et al. 2021). There are growing number of evidence demonstrating the requisite of autophagy and vesicle trafficking to *F. graminearum* growth and pathogenicity (Li et al. 2017; Wu et al. 2023; Yuan et al. 2022).

In the rice blast fungus *M. oryzae*, conidiation and polarized growth have been shown to be directly linked to endocytosis via ADP-ribosylation factor 6 (Arf6)-mediated vesicle trafficking (Zhu et al. 2016). Also, the Arf-GAP (GTPase activating protein) MoGlo3 regulates COPI-mediated trafficking, endocytosis, stress tolerance, pathogenicity and development of the blast fungus (Zhang et al. 2017). A recent study established that the absence of MoSwa2, a protein that plays a key role in regulating the secretory pathway by uncoating of COPII in *M. oryzae*, perturbs *M. oryzae* virulence, suggesting a link between vesicle trafficking and pathogenesis of the hemibiotrophic fungus (Liu et al. 2021). Moreover, the ER-derived vesicle (ERV) proteins MoErv14 and MoErv29 were shown to be indispensable for vesicle-mediated secretion of many proteins, and are also critical for the development and virulence of *M. oryzae* (Qian et al. 2022, 2023). Generally, the active growth sites of fungal pathogens receive plethora of vesicles containing different cargos for different functions. The pathogenesis of phytopathogens depends on vesicle-mediated extracellular delivery of virulence factors, which is regulated by a number of vesicle trafficking regulators such as the Qc-SNARE protein MoSyn8 and protein phosphatase 1/Vps45 (Bryant and James 2003; Qi et al. 2016). Fungal pathogens also internalize EVs coming either from their hosts or from other fungi and the cargos of such vesicles could modulate their growths and virulence. Depending on their contents, EVs can serve protective and/or pathogenic functions. However, the mechanism of EV cargo selection for a particular function remains unknown. Pathogenic fungi and oomycetes also secrete small RNA-containing vesicles to infection sites for delivery into the host to shut down the host immune responses via RNA interference (U Stotz et al. 2022).

Emerging evidences suggest that pathogenic fungal EVs are determinants of their virulence as a number of bioactive molecules that relate to the fungal virulence and metabolism have been identified as cargos in such EVs (Bleackley et al. 2019b; Herkert et al. 2019). However, very little is known so far about the link between EVs released by phytopathogenic fungi and the fungal virulence. Be that as it may, some phytopathogenic fungi have been shown to release virulence responsive EVs (Bleackley et al. 2019a). Phytopathogenic fungi generally release ILVs containing many different cargos during interactions with their host plants. *M. oryzae* and *Phytophthora infestans* were independently demonstrated to deliver effector molecules to their hosts using unconventional secretion pathway (Liu et al. 2021; S., Wang et al. 2018a, b). This pathway largely depends on vesicle transport from biotrophic interfacial complex (BIC)/haustorium formed by the two fungi, respectively, to their hosts.

**Fig. 3:**
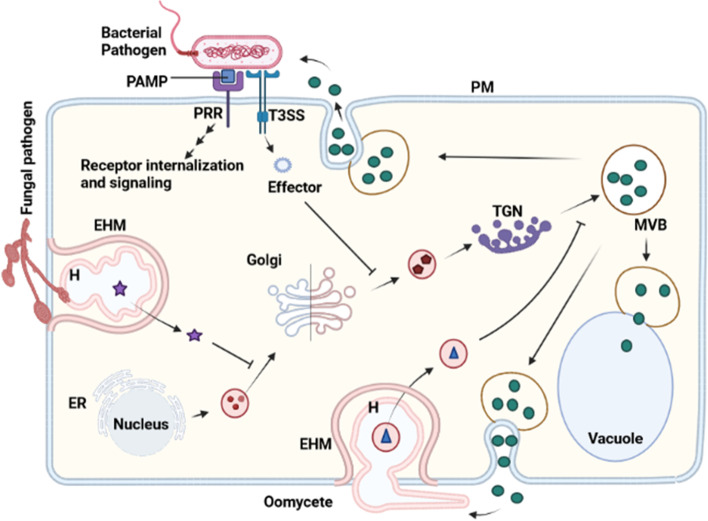
Vesicle trafficking-dependent plant immune responses and their suppression by phytopathogens during interaction. Upon identification of bacterial PAMPs, PRRs are internalized to trigger downstream signaling which leads to immune responses by the host. The host counterattacks include the synthesis, packaging and release of antimicrobials to the infection site. The pathogen releases effector substances which block some vesicle trafficking pathways to render the host susceptible and prevent counter-responses. Oomycetes and fungal pathogens also release virulence factors through the haustoria for same purposes. *PRR:* Pattern recognition receptor, *PAMP:* Pathogen associated molecular pattern, *H:* haustoria, *EHM:* Extra haustorial membrane, *ER:* Endoplasmic reticulum, *TGN:* Trans-Golgi network, *MVB:* Multivesicular body, *T3SS:* Type 3 secretion system, *PM:* Plasma membrane

EVs may contain not only protein cargos but also some other molecules like carbohydrates, lipids and nucleic acids such as DNA, RNA or even non-coding RNA, and these cargos are protected from proteases and nucleases by the vesicle membrane after release into the extracellular space Bleackley et al. 2019a). Although not proven in plants/plan fungal pathogens, animal exosomes could contain mRNAs that are translated into proteins after delivery to a target cell/organism (Colombo et al. 2014). During plant-fungal pathogen interaction, both the host plants and the fungal pathogen exchange vesicles containing cargos that modulate each other’s gene expression, suppress immunity, inhibit important pathways (e.g. vesicle trafficking pathway) or exert antimicrobial activity, as the case may be (Wang and Dean 2020). Phytopathogenic fungi package and release virulence factors in EVs for delivery to infection sites to manipulate their hosts and establish successful infection. Effector substances from phytopathogenic fungi and oomycetes are released through their haustoria during interaction with the hosts. The oomycete *Phytophthora infestans* uses the haustoria to release the effector protein AVRblb2 into its hosts to block the trafficking of the host C14 papain-like cysteine protease to the apoplast (Wang et al. 2017). Some effector molecules block the host vesicle trafficking pathways in favor of the pathogens (Fig. 3) and most effector proteins from phytopathogens directly or indirectly target and suppress the host plant’s PTI (Hetmann and Kowalczyk 2019). Vesicle-mediated recycling of PEN1 between the PM and the cytoplasm of plants is indispensable for their basal immunity. The fungal pathogen *Golovinomyces orontii* targets and blocks the PEN1 recycling pathway for effective proliferation in Arabidopsis (Kim et al. 2016). Effectors from some phytopathogenic fungi are restricted by certain specialized plant-derived membranes other than the EHM, such as extra-invasive hyphal membrane (EIHM) due to infection by fungi belonging to the order Colletotrichum (Shimada et al. 2019). The sunflower pathogen *Sclerotinia sclerotiorum* was shown to take up EVs from the host plant, and this negatively affects the fungal invasive growth in the host (Regente et al. 2017).

## Roles of vesicles trafficking in plant development and immunity against fungal pathogens

The vesicle trafficking pathways in plants play many important roles including, but not limited to, prevention of pathogen entry, cell–cell communication, growth and development, endomembrane system reprogramming, delivery of important biomolecules to targeted sites (Suharta et al. 2021). The ER play a key role in ensuring a continuous vesicle trafficking process, especially that of the secretory pathway. Most important membrane proteins, such as the PRRs, are synthesized in the ER, packaged in vesicles at the Golgi and transported to the PM where they exert their biological functions, suggesting a link between vesicle trafficking and pathogen detection (Ruano and Scheuring 2020; Underwood 2016). Following endocytosis, these PM-localized proteins are internalized in endocytic vesicles and delivered to the endosomes where cargos are sorted and channeled to other destinations. Internalization of PRRs due to pathogen attack triggers signal transduction that leads to immune responses (Gu et al. 2017). The internalized PM proteins are sorted and returned back to the PM through MVBs. After activation and subsequent internalization of FLS2, a well-studied plant PRR that detects flg22 (a notable bacterial PAMP) and promotes plant immunity against bacterial pathogens, the protein is recycled back to the PM via MVB-mediated trafficking. The sorting and trafficking of these vesicles are mediated by different motor proteins such as the retromer complex, dynamins and kinesins (Abubakar et al. 2017; Heucken and Ivanov 2018; Paez Valencia et al. 2016). However, how these vesicles selected by the different molecular motors in different trafficking pathways are still unclear. Also, how transport vesicles are targeted to a particular PM domain during secretion awaits future research.

Plant cells also release EVs into the extracellular space for specific purposes (Bhandari and Brandizzi 2020). This ensures timely and effective responses to invading pathogens. Almost all steps of plant immune responses to pathogen attack involve targeted vesicle transport, including pathogen recognition, secretion of antimicrobials, ROS generation, and so on (Gu et al. 2017; Ruano and Scheuring 2020). Plants synthesize pathogenesis-related proteins, lipids, nucleic acids and other metabolites, which are packaged in transport vesicles and delivered to pathogen infection sites for antimicrobial activity (Gu et al. 2017; Tsatsaronis et al. 2018). Arabidopsis release EVs containing sRNA at *Botrytis cinerea* infection sites, which, when taken up, target and silence the fungal pathogenesis-related genes (Cai et al. 2018). Generally, under pathogen attack, plants release EVs into the extracellular space as part of their immune responses (U Stotz et al. 2022). In fact, plant EVs are known to contain bioactive compounds against pathogenic fungi (Boevink 2017; Regente et al. 2017). Sunflower releases cell wall degrading enzymes against *B. cinerea* (U Stotz et al. 2022). *Arabidopsis* delivers biotic and abiotic tress-response proteins in EVs to extracellular environment (Rutter and Innes 2017). PEN1/SYP121 and PEN3/PDR8 were observed to accumulate at infection sites following attempted penetration by powdery mildew fungi (Pakuła et al. 2023; Wu et al. 2020). Tetraspanin-rich vesicles were also shown to concentrate at infection sites; tetraspanins are important for membrane trafficking and signaling in plants (Cai et al. 2018; Reimann et al. 2017).

Oomycetes and fungal phytopathogens form haustoria through which they release effector substances to subvert the plant immune responses whereas bacterial pathogens use their secretion systems to release the virulence factors (Fig. 3). To restrict pathogen influence, plants surrounds the hautorial membrane with EHM the formation of which requires transport vesicles delivery (to the haustorial neck) rather than PM invagination (Berkey et al. 2017; U Stotz et al. 2022). Arabidopsis *ARA6* (*RAB5* homologue) product was also shown to localize to the EHM; some host proteins are however selectively excluded on this defense barrier ( Inada et al. 2016). Vesicle-Associated Membrane Proteins (VAMPs) are very crucial in plants’ counter responses to fungal pathogens. The post-invasive resistance protein RPW8.2 is delivered to the EHM from TGN in VAMP722-positive vesicles and the EHM localization of this protein enhances Arabidopsis resistance to powdery mildew pathogen (Park et al. 2018). Furthermore, VAMP721-dependent delivery of cell wall modification enzymes in Arabidopsis is critical in preventing pathogen penetration at the EHM (Uemura et al. 2019). Similarly, in rice, VAMP714-positive membranes were shown to localize to *M. oryzae* infection sites, suggesting a role in blast resistance (Sugano et al. 2016).

## Conclusion and prospects

Plants interact with different microorganisms and the interactions can be mutualistic or pathogenic. This review focuses on the exchange of cargo-containing vesicles between plants and phytopathogenic fungi. Findings from previous studies clearly demonstrated that both plants and their invading fungal pathogens utilize vesicle trafficking for different purposes. Both organisms need vesicle trafficking for their normal physiologic activities. Additionally, the pathogenesis of fungal pathogens largely depends on the delivery of pathogenesis-related molecules packed in membrane-bound vesicles. Most of these pathogens lose their pathogenicity when their vesicle trafficking pathways are perturbed. In other words, the pathogens package virulence factors in transport vesicles and release them to the hosts for successful colonization. Similarly, hosts also use their vesicle trafficking pathways to defend themselves against the pathogen attacks. They do not only neutralize the virulence substances from the pathogens but also block the pathogens’ vesicle trafficking pathways. However, a number of knowledge gaps in our understanding of the use of transport vesicles in plant-fungal pathogen interactions still exists. What are the factors that determine cargo selection for a kind of vesicle? What determines the specific pathway for MVB transportation? Can EVs be released for plants/pathogenic fungi detection or are they only released post detection? These and many related questions await future investigations. Understanding these will provide new horizon in plant-fungus warfare and plant disease management.

## Data Availability

All the data supporting the claims contained in this manuscript are provided in the submission and can be shared publicly after acceptance of the manuscript for publication by Stress Biology.
